# Serum biomarkers of *Burkholderia mallei* infection elucidated by proteomic imaging of skin and lung abscesses

**DOI:** 10.1186/s12014-015-9079-4

**Published:** 2015-03-10

**Authors:** Trevor G Glaros, Candace D Blancett, Todd M Bell, Mohan Natesan, Robert G Ulrich

**Affiliations:** Molecular and Translational Sciences, USAMRIID, Frederick, 21702 MD USA; Pathology, U.S. Army Medical Research Institute of Infectious Diseases, Frederick, 21702 MD USA

**Keywords:** Imaging mass spectrometry, Biomarker, *Burkholderia mallei*, *Burkholderia pseudomallei*, Laser capture microdissection, LC-MS/MS, Protein microarray, Glanders, Melioidosis, GroEL, Calprotectin, Formalin-fixed paraffin embedded tissue, FFPE

## Abstract

**Background:**

The bacterium *Burkholderia mallei* is the etiological agent of glanders, a highly contagious, often fatal zoonotic infectious disease that is also a biodefense concern. Clinical laboratory assays that analyze blood or other biological fluids are the highest priority because these specimens can be collected with minimal risk to the patient. However, progress in developing sensitive assays for monitoring *B. mallei* infection is hampered by a shortage of useful biomarkers.

**Results:**

Reasoning that there should be a strong correlation between the proteomes of infected tissues and circulating serum, we employed imaging mass spectrometry (IMS) of thin-sectioned tissues from *Chlorocebus aethiops* (African green) monkeys infected with *B. mallei* to localize host and pathogen proteins that were associated with abscesses. Using laser-capture microdissection of specific regions identified by IMS and histology within the tissue sections, a more extensive proteomic analysis was performed by a technique that combined the physical separation capabilities of liquid chromatography (LC) with the sensitive mass analysis capabilities of mass spectrometry (LC-MS/MS). By examining standard formalin-fixed, paraffin-embedded tissue sections, this strategy resulted in the identification of several proteins that were associated with lung and skin abscesses, including the host protein calprotectin and the pathogen protein GroEL. Elevated levels of calprotectin detected by ELISA and antibody responses to GroEL, measured by a microarray of the bacterial proteome, were subsequently detected in the sera of *C. aethiops, Macaca mulatta,* and *Macaca fascicularis* primates infected with *B. mallei*.

**Conclusions:**

Our results demonstrate that a combination of multidimensional MS analysis of traditional histology specimens with high-content protein microarrays can be used to discover lead pairs of host-pathogen biomarkers of infection that are identifiable in biological fluids.

**Electronic supplementary material:**

The online version of this article (doi:10.1186/s12014-015-9079-4) contains supplementary material, which is available to authorized users.

## Background

Elevated levels of pathogen-specific antibodies and antigens are indicators of current or recent infection, while perturbations of other serum proteins can illuminate disease progression and recovery. The identification of these classes of critical biomarkers in biological fluids obtainable by non-invasive means is generally performed by trial and error. Thus, progress in diagnosis and treatment of many infectious diseases will greatly benefit from higher throughput methods for the discovery of biomarkers associated with immune responses to infection. *Burkholderia mallei* is a gram-negative bacterium that causes glanders [1], a disease primarily affecting *Equidae,* most commonly horses, which may transmit to humans by direct contact with infected animals [2]. Although human infections are rare, *B. mallei* can enter the body through the eyes, nose, mouth, or breaks in the skin [3]. Contact with the skin may lead to a localized infection, while inhalation of aerosolized *B. mallei* can lead to acute or chronic infections that have a mortality rate greater than 50% even when treated with antibiotics [4,5]. Besides the veterinary disease, there is a public health concern for potential human infection from acts of bioterrorism. There are no vaccines for glanders, few reliable diagnostic tests and little information concerning correlates of immunity. Glanders is diagnosed in the laboratory by isolating *B. mallei* from blood, sputum, urine or skin lesions. Limited use of PCR based tests [6], complement fixation and agglutination assays [7] were also reported.

Mass spectrometry based on matrix-assisted laser desorption ionization (MALDI) utilizes a reactive matrix that is co-crystalized with analytes to enhance ionization induced by UV laser activation energy for measurement of ion masses by time of flight (TOF). MALDI imaging mass spectrometry (IMS) is a specific approach that is used to map the spatial distribution of analytes in tissue sections at a level of detail that is not possible by other methods [8]. To perform IMS, tissue sections are coated with matrix on conductive slides, and analyzed by MALDI TOF-TOF. Spectra are collected in MS reflector mode by a defined laser raster pattern, with each laser pulse generating a single image pixel. An ion intensity map is used to visualize the relative abundance and spatial distribution of analytes *in situ*. Digital overlays of MS spectral maps with standard histology images can then be used to co-localize proteomic data within tissue features observable by microscopy. Despite many technical obstacles that need to be overcome in this developing field [9], IMS has been successfully used to study the tissue distribution of lipids [10], proteins [11], peptides [12], and pharmaceuticals [13]. Contrary to other imaging techniques, such as immunohistochemistry (IHC) and fluorescent microscopy, IMS does not require any target or tissue specific reagents and is capable of simultaneously analyzing hundreds of molecular features [14]. Tissue specimens are most commonly fixed with formalin and embedded in paraffin (FFPE) for microscopic analysis. Formalin-fixation preserves tissue structure by cross-linking proteins and also serves to inactivate infectious agents. Unfortunately, this standard method of tissue preparation is detrimental to IMS due to the difficulty in obtaining tryptic digests of formalin cross-linked proteins that provide interpretable MS2 fragmentation data [15]. Formalin fixation can be partially reversed [16] by heating the tissue to temperatures up to 100°C, thus making it possible to use FFPE tissue for IMS. However, there are few reported IMS studies that have used FFPE tissue processed for antigen retrieval, and thus the limitations of this approach are not clear. We used IMS to examine FFPE tissues from *Chlorocebus aethiops* monkeys infected with *B. mallei*. The objective was to identify the specific pathogen and host-response biomarkers within FFPE tissues that were also detectable in serum. Utilizing antigens identified through tissue analysis with IMS, we also examined serum antibody responses by a focused proteome microarray comprised of *Burkholderia* proteins. Our study demonstrates that IMS can be used to recover proteomics data from formalin-fixed tissue and that these results can be used to identify serum biomarkers of infection.

## Results

### Imaging mass spectrometry

**Figure 1:**
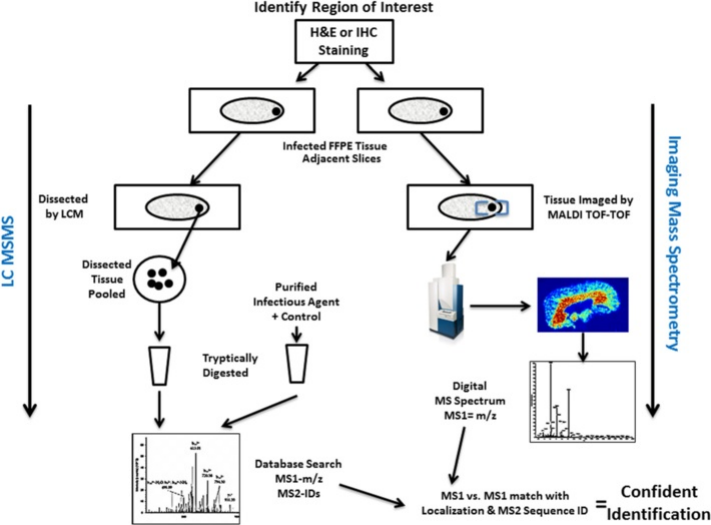
**Overview of the proteomics strategy for biomarker discovery.** Abscesses of infection were microscopically identified in thin-sectioned tissues (formalin-fixed, embedded in paraffin) by histology (H&E stained) and localization of bacteria by specific antibody (IHC). The tissue sections were next examined by imaging mass spectrometry (IMS) to identify analyte masses that were localized to the selected regions of interest. Using laser-capture microdissection of select regions of the tissue sections identified by IMS and histology, a more extensive proteomic analysis could then be performed by a technique that combines the physical separation capabilities of liquid chromatography (LC) with the sensitive mass analysis capabilities of mass spectrometry (LC-MS/MS). Finally, the LC-MS/MS data was compared to masses observed by IMS for highest confidence in biomarker identification.

**Figure 2:**
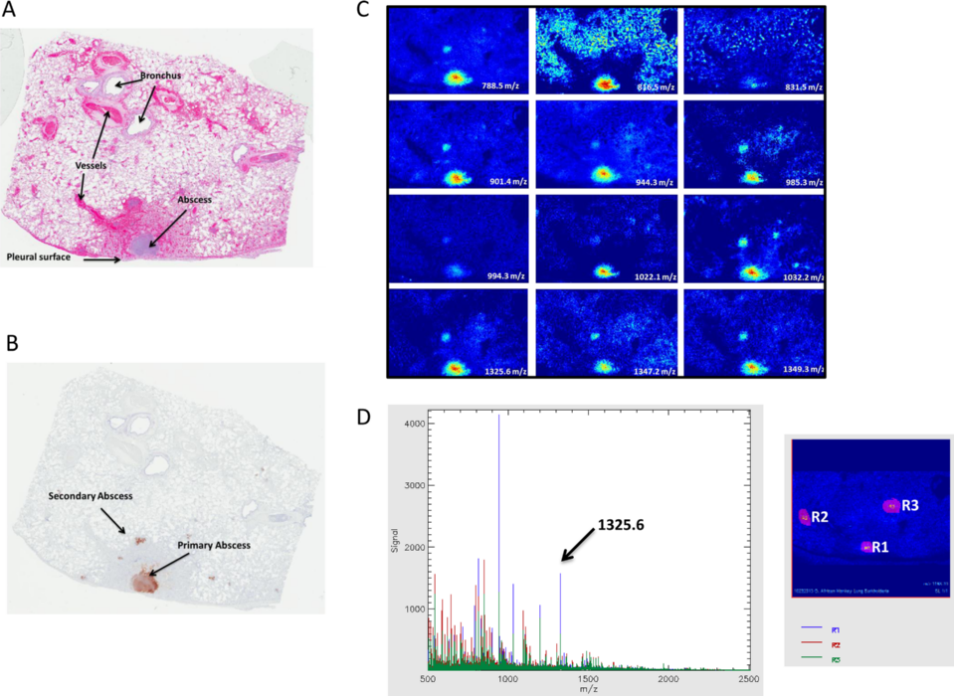
**Histopathology and imaging of** ***B. mallei*** **infection in the lung of a** ***Chlorocebus aethiops*** **monkey.** Tissues were collected at the time of death. **(A)** Histological image (H&E stain) of infected lung tissue. **(B)** Immunohistochemistry using *B. mallei-*specific antibody to visualize bacteria. **(C)** Collection of ion intensity maps, spatial resolution of 75 μm, localized to site of infection. Signal intensity images are presented as a blue (lowest) to red (highest). **(D)** Mass spectrum extracted from the inflammatory abscess (R1-Blue) overlaid with the mass spectrum from two background regions (R2-Red and R3-Green) normalized to total ion current. The ion 1325.6 m/z is more abundant in the abscess compared to the background.

An overview of the experimental process that was used to link proteomic data from infected tissues to biomarkers detectable in serum is shown in Figure 1. Lung and skin tissues procured during necropsy of *Chlorocebus aethiops* monkeys that succumbed to aerosol infection by *B. mallei*, as well as non-infected controls, were examined for inflammation and bacterial burden associated with glanders. Evidence of the targeted bacteria within processed tissues (FFPE) was obtained from microscopic observation of H&E (Figure 2A) and anti-*Burkholderia* IHC stained slides (Figure 2B). Because the respiratory tract was the primary route of infection, we first examined lung tissue that contained an abscess with *B. mallei* observable by IHC (Figure 2B). Microscopic analysis of the H&E section showed an abscess composed of cellular debris, numerous degenerate neutrophils, and macrophages that contained abundant intracytoplasmic basophilic material composed of rod shaped bacteria. The area immediately surrounding the abscess consisted of numerous macrophages, small blood vessels, elongated fibroblasts, and collagen.

A 5 μm tissue section that was adjacent to the infected specimen identified by histology was processed for MALDI-IMS. The IMS results revealed 12 potential ions that were localized to the areas known to contain *B. mallei* and had an increased signal compared to the control background (Figure 2C). All of these ions were increased within the central abscess and some were also elevated in the secondary abscess (Figure 2B,C). To build a database of potential ions from *B. mallei*, a bacterial whole cell lysate was digested and analyzed by LC-MS/MS. This analysis resulted in the generation of a *B. mallei* specific library containing nearly 500 proteins and more than 1300 unique peptides. Peptides from the most abundant proteins were compared to the masses presented in Figure 2C. This strategy resulted in a tentative match to the peptide YVASGMNPMDLK from GroEL, with a mass of 1325.6 m/z. The mass spectrum from a spot within the abscess clearly shows the presence of a 1325.6 m/z analyte compared to the background (Figure 2D).

### Laser capture microdissection and LC-MS/MS analysis

**Figure 3:**
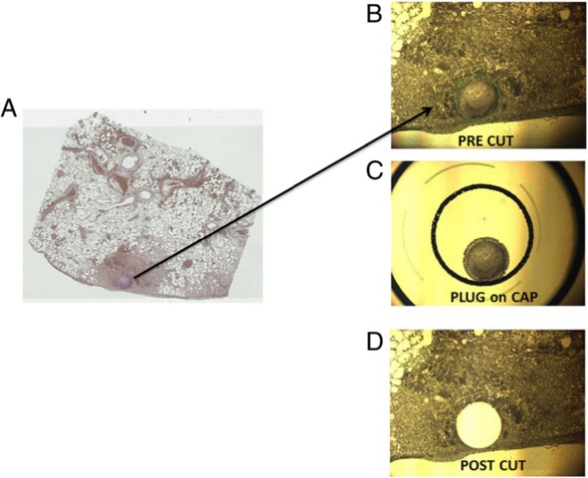
**Laser-capture tissue microdissection. (A)** Diseased lung tissue was formalin-fixed, embedded in paraffin and stained with Mayer’s hematoxylin. **(B)** Microscopic image (2X magnification) indicating the region prior to microdissection (green circle). **(C)** Microdissected target area of the tissue section captured on an adhesive cap. **(D)** Tissue section showing the removed microdissected area.

**Figure 4:**
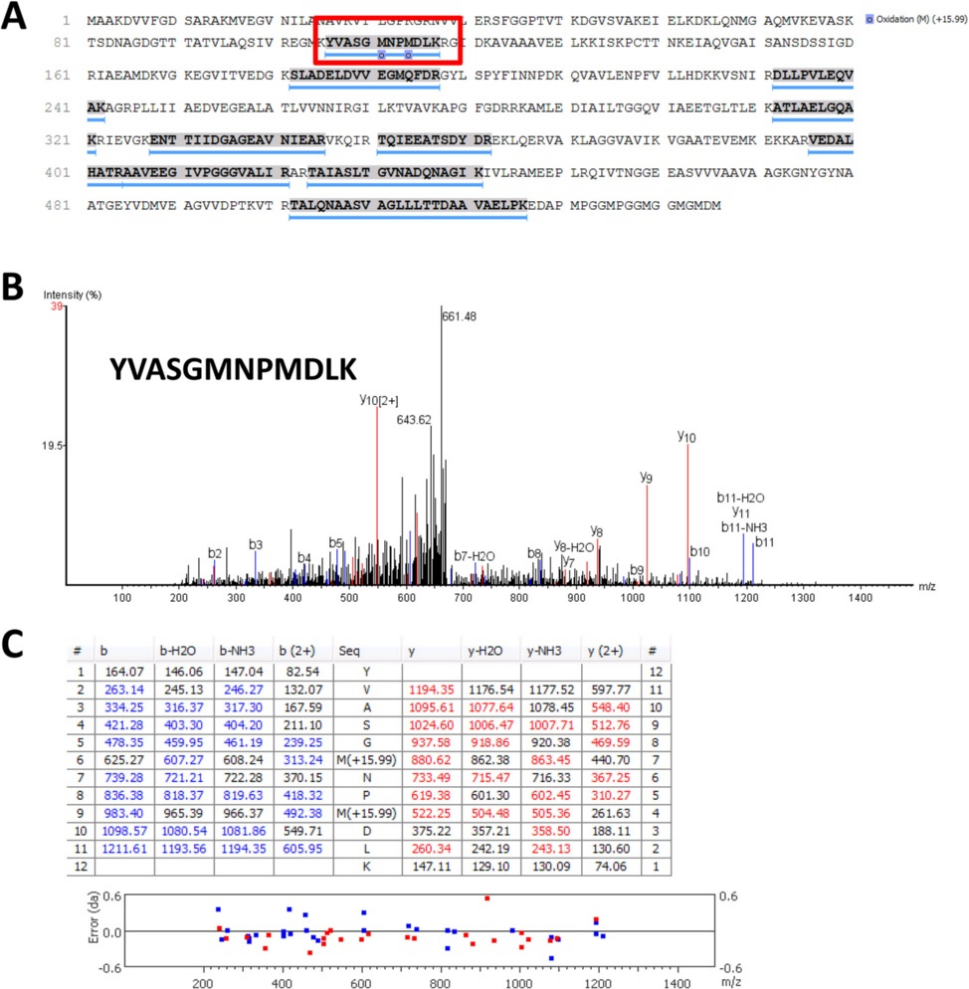
**Identification of the bacterial protein GroEL from infected** ***Chlorocebus aethiops*** **lung tissue. (A)** Total protein coverage attained for GroEL from LC-MS/MS data generated from the LCM tryptic digestion. The peptide sequence YVASGMNPMDLK is boxed in red. **(B)** CID MSMS spectra of 1356.6, which matched GroEL peptide YVASGMNPMDLK, with a mass deviation of −1.8 ppm. **(C)** Fragment b and y ions that matched predicted b and y ions are highlighted in blue and red, respectively. Graphed below is the observed mass deviation from predicted mass for each matched fragment ion.

**Figure 5:**
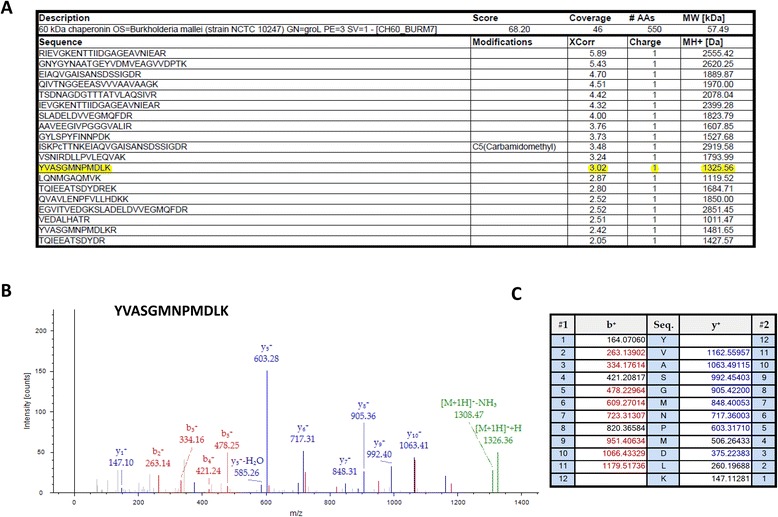
**Recombinant** ***B. mallei*** **GroEL analyzed by MALDI TOF-TOF. (A)** Total protein coverage of GroEL was 46 percent. YVASGMNPMDLK was observed by MALDI MSMS without methionine oxidation. (**B** and **C**) CID MSMS spectra of YVASGMNPMDLK with a mass of 1325.6 m/z. Fragment b and y ions that matched predicted b and y ions are highlighted in blue and red respectively.

Because the bacterial proteins within the histology slides were likely to be only a small fraction of the total protein present in the tissue section, we used laser capture microdissection (Figure 3) to isolate material associated with only the bacterial abscess and a minimal amount of surrounding host tissue. Pooled microdissections from five serial sections were examined by LC-MS/MS (Figure 4), resulting in the identification of several peptides from GroEL (Figure 4A), including the peptide sequence YVASGMNPMDLK observed in the whole cell lysate (Figure 4B,C). Although the peptide was a tentative sequence match with the sequence from the *B. mallei* whole cell lysate, the MS1 mass (1356.6 m/z) was not identical due to oxidation of the methionine residues. To determine if methionine oxidation was an artifact of tissue preparation and analysis by LC-MS/MS, recombinant GroEL was tryptically digested for analysis by MALDI TOF TOF. Our results with the recombinant GroEL revealed the peptide YVASGMNPMDLK with no methionine oxidation and a MS1 mass of 1325.6 m/z (Figure 5). Taken together, this data indicated that the ion mass of 1325.6 detected by IMS within infected lung tissue was indeed a peptide fragment from *B. mallei* GroEL. In addition to GroEL from *B. mallei*, 480 other host proteins (Additional file 1: Table S1) were identified in the abscess. To maximize identification of potential host proteins, the MS2 spectra were searched against the curated Swiss-prot database for Cercopithecidae (Old World monkeys).

### Associations between serum and tissue markers

**Figure 6:**
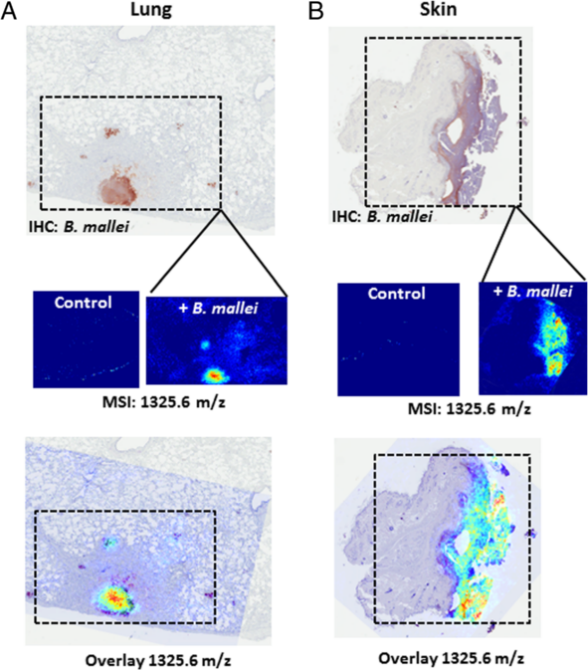
**Histopathology comparison of the 1325.6 IMS in both diseased lung (A) and skin (B) from individual** ***Chlorocebus aethiops*** **monkeys.** Digital microscopic images obtained from immunohistochemical identification of *B. mallei* in diseased lung and skin sections were overlaid with IMS images for ion 1325.6. Digital images of healthy control tissue sections of lung and skin were used as negative controls.

**Figure 7:**
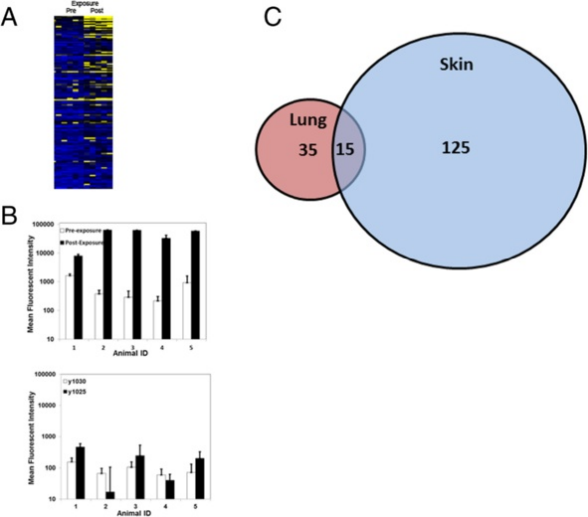
**Differentially detected host and pathogen proteins of** ***B. mallei*** **infection. (A)** Heatmap of IgG interactions from *Chlorocebus aethiops* sera (individuals in columns) with microarrayed *B. mallei* proteins (in rows). **(B)** Antibody responses (±SD) to GroEL in comparison to controls were significantly elevated (p ≤ 0.05) in serum collected after infection with *B. mallei* (middle panel) from four *Chlorocebus aethiops* monkeys that succumbed to infection, on days 5 (ID 1), 11 (ID 2), 13 (ID 3), 14 (ID 4); and in serum collected on 28 from one monkey that recovered from infection (ID 5). There were no measurable antibody responses from any of the monkeys to the control proteins y1030 and y1025 from the closely related bacterium *Yersinia pestis* (lower panel). **(C)** Venn diagram representing the number of qualitative host protein changes observed in the lung (35), the skin (125), and overlapping (15). The proteins overlapping in the Venn diagram are presented in Table 1.

Microsections of infected skin from another *Chlorocebus aethiops* monkey (not the same subject from which the lung biopsy was obtained) was examined to confirm that the GroEL peptide was more than just a tissue-specific marker. For both lung and skin tissue, the 1325.6 m/z analyte co-localized with *B. mallei* in the IMS and IHC image overlay shown in Figure 6. These results suggested that GroEL was present at a distal site of infection as well as the primary portal of entry. As there is no experimentally determined 3-dimensional structure for the *B. mallei* protein, the GroEL tertiary structure was predicted (100% confidence) using Phyre2 [17], based upon the published crystal structure of GroEL from *E. coli* with a 76% sequence identity [18]. The peptide sequence YVASGMNPMDLK is located between two adjacent alpha helixes on the protein surface (data not shown), and is likely to be readily accessible to trypsin. Because our objective was also to identify pathogen biomarkers within FFPE tissues that were associated with host serological responses to infection, we also examined antibody response to GroEL. A microarray comprised of approximately 300 recombinant proteins from *B. mallei*, including GroEL as well as control proteins from the gram-negative pathogen *Yersinia pestis*, was used to measure serological immune responses to infection. The recombinant proteins were printed in 120 μm microarray spots on slides coated with a thin layer of nitrocellulose, and antibody interactions with the immobilized antigens were measured (Figure 7A) using previously described methods [19,20]. We observed that *Chlorocebus aethiops* monkeys infected with *B. mallei* exhibited a 10–100 fold increase in antibodies specific to GroEL compared to antibody responses against the control proteins y1030 and y1025 from *Y. pestis* (Figure 7B).

To delineate the qualitative host protein changes we compared host protein abundances in the lesion (positive IHC stain) to a control area in the same tissue (negative IHC stain). This comparison resulted in the detection of 35 host protein changes in the lung and 125 host protein changes in the skin (Figure 7C). To strengthen the confidence in host protein changes, only proteins identified in both the skin and lung lesion were considered. This analysis resulted in 15 proteins (Table 1) that were qualitatively different when compared to the respective controls (Figure 7B). We selected calprotectin for more detailed analysis because of a previously reported association with other gram-negative infections [11]. Sera were obtained from three nonhuman primates species (*Chlorocebus aethiops, Macaca mulatta,* and *Macaca fascicularis*) infected with *B. mallei*, and calprotectin levels were measured by ELISA. Calprotectin was significantly elevated in sera from all infected individuals compared to noninfected controls (Table 2). Our results indicated that elevated levels of the host protein calprotectin within infected skin and lung correlated with increased serum levels and that this biomarker of infection was detectable in three primate species. We noted that bacteremia occurred in only two of the six infected *Chlorocebus aethiops*, while five individuals later succumbed to infection, suggesting that elevated serum calprotectin was a better indicator of active infection.

**Table 1:** **Relative abundance of specific host proteins from infected lung and skin tissues**

		**Diseased fold-change** ^**1**^		
**Description**	**General function**	**Accession**	**Lung**	**Skin**
Calprotectin	Innate immunity	F6QJD8	+255	+
78 kDa glucose-regulated protein	Adaptive immunity	F7C3R1	+14	+
Alpha-1-acid glycoprotein 1	Acute phase protein	G7PRL2	+	+10
Alpha-1-antichymotrypsin	Acute phase protein	F6SL79	+	+8
C3 and PZP-like alpha-2- macroglobulin domain-containing protein 1	Inflammation induced	G7PYU9	+20	+256
Calreticulin (CRP55)	Calcium homeostasis	F7FMQ2	+67	+
Endoplasmin	Immune chaperone	F7EZT6	+10	+256
Erythrocyte band 7 integral membrane protein isoform a	Red blood cell protein	F7HP19	+255	+
Fibrinogen alpha chain	Clotting	F6UZ60	+25	+203
Hemoglobin alpha chain	Red blood cell protein	G7Q017	−20	+12
Hemoglobin subunit beta	Red blood cell protein	F7APQ4	+3	+
Leukocyte elastase inhibitor	Innate immunity	G7P4B6	+27	+
Purine nucleoside phosphorylase (Fragment)	Purine metabolism	G8F5P6	+10	+
Scinderin	Secretory pathway	F6T8Z7	+	+
Serum amyloid A protein	Acute phase protein	F7DY68	+63	+4
Superoxide dismutase [Mn] mitochondrial	Innate immunity	Q8HXP2	+75	+26

^1^Label-free quantitative analysis of proteins analyzed by LC-MS/MS. Data labeled with only a “+” symbol denotes proteins that were detected in the infected animals but not in the uninfected control under these experimental conditions.

**Table 2:** **Serum calprotectin [μg/ml]**

***Chlorocebus aethiops***	**Collection day**	**Pre-exposure**	**Post-exposure** ^**1**^
1	5	1.45	>12.5*
2	11	1.09	>12.5*
3	13	0.70	>12.5*
4	14	1.89	7.27*
5^2^	28	2.27	10.55*
***Macaca mulatta***		**Pre-exposure**	**Post-exposure**
1	28	2.8	>25
2	28	3.2	16.8*
3	28	1.9	23.3*
4	28	1.5	21.9*
5	28	5.9	12.7*
***Macaca fascicularis***		**Pre-exposure**	**Post-exposure**
1	28	8.3	17.8
2	28	1.0	9.0*
3	28	1.5	6.4*
4	28	2.1	7.2*

^1^Calprotectin concentration determined by ELISA, averaging the absorbance (450nm) of at least two technical replicates per primate and comparison to a standard curve.

^2^This was the only individual of this species surviving infection by aerosol exposure to *B. mallei*.

*: p ≤ 0.05.

## Discussion

We used MALDI IMS combined with optical microscopy to examine proteomic changes in tissues from *Chlorocebus aethiops* monkeys infected with *B. mallei*, and to identify correlations between biomarkers from solid tissue and serum. Abundant protein-derived ions were observed in lung and skin tissues of subjects infected by aerosol exposure to the virulent bacterial pathogen. By coupling MALDI IMS, laser capture microdissection, and LC-MS/MS we were able to identify bacterial GroEL as a specific biomarker of *B. mallei* infection. Two-dimensional intensity distributions of detectable analytes indicated that GroEL was only associated with solid tissue sites harboring bacteria. Protein microarray analysis enabled us to observe robust antibody responses to GroEL in all infected *Chlorocebus aethiops* monkeys, allowing us to connect a pathogen marker detectable in tissue with host responses readily detected in serum. In addition, qualitative analysis of host proteomic changes revealed perturbations in protein compositions of the infected lesions that were consistent with inflammation and active immune responses. In particular, we further examined calprotectin and found that levels were significantly elevated within the serum as well as infected solid tissues of infected primates. Calprotectin is a cytosolic protein of neutrophils and is a clinical marker for intestinal inflammation [21]. Although we only examined calprotectin in detail, it is possible that other host proteins within the skin and lung abscesses were also elevated in serum as a result of *B. mallei* infection and may be equally important disease biomarkers. Thus, standard FFPE tissue sections processed for optical microscopy were used as primary sources of proteomic data to facilitate the identification of the pathogen protein GroEL and the host response protein calprotectin as paired markers of *B. mallei* infection. Taken together, our results indicate that IMS can be used for discovery of biomarkers that are relevant to infectious diseases. Although tissues from an animal model of glanders were used for our study, it is also conceivable that human biopsy material could be used as a starting point for biomarker discovery by the method we describe.

Considering the abundance, safety and availability of FFPE tissue, the utility of combining MALDI IMS with LC-MS/MS samples enriched by laser capture microdissection of histology specimens is an important advancement for biomarker discovery demonstrated by our study. Presently, the dynamic range of MALDI IMS and the ability to process FFPE tissue are significant limiting factors [14]. Formalin-fixation induces diverse chemical modifications of peptides, including the formation of methylol groups, Schiff bases, and methylene bridges [15]. Tyrosine, arginine, and lysine residues are most reactive, although other amino acid side chains are also susceptible to formalin modification, which is also highly dependent on protein tertiary structure [22]. The GroEL peptide YVASGMNPMDLK is located in a region that is likely to be solvent accessible, while the most reactive side chains (tyrosine and lysine) are buried within two separate alpha helices [17]. Since formalin treatment occurs prior to heating or any other tissue manipulation, it is possible that the GroEL tyrosine and lysine side chains were buried and not freely available for chemical modification in the tissues examined. However, we obtained proteolytic fragments of GroEL from infected tissues that were treated with a heat-induced antigen retrieval process prior to trypsinization. Alternative chemical or enzymatic approaches may be useful to obtain peptides from FFPE tissues that are identifiable by MS, and this is a subject area that should be systematically examined in future studies.

We considered the significance of our initial observations on identifying biomarkers of *B. mallei* infection. GroEL is a very abundant and conserved protein of gram-negative bacteria that was previously reported to be highly immunogenic in human and non-human primates [23]. In addition to its potential use as a diagnostic, GroEL is being explored for use in vaccines against tuberculosis, brucellosis, and yersiniosis bacterial infections [24-26]. Further, GroEL may be expressed on the outer cellular membrane of the closely related *B. pseudomallei*, and is actively secreted by *Bartonella bacilliformis* [27,28]. The peptide sequence we detected by MS is specific to the genus *Burkholderia*, and thus may have utility in distinguishing between infections caused by *Burkholderia* spp. and related gram-negative pathogens, for example *Pseudomonas aeruginosa* and *Klebsiella pneumoniae*. Discriminating *B. mallei* infections from those caused by the very closely related human pathogen *B. pseudomallei* will require inclusion of additional species-specific biomarkers. For the case of infections caused by strains of *B. pseudomallei*, which exhibit substantial genetic variability and contrasting pathologies, a more extensive analysis of additional tissues will be required before the final utility of the specific markers we describe can be determined. For example, a recent genetic analysis by Sarovich and coworkers identified a *B. mallei*-like actin polymerization bimA(Bm) gene that was strongly linked to neurological disease and a filamentous hemaglutinin gene, fhaB3, that was associated with positive blood cultures but negatively correlated with localized skin lesions without sepsis [29]. The results we present, though targeted specifically to glanders, illustrate a general approach for discovery of biomarkers that are relevant to other infectious diseases.

## Conclusions

We used imaging MS coupled with laser capture microdissection and LC-MS/MS to identify disease-associated proteins that were present in FFPE tissues from African green monkeys infected by aerosol exposure to *B. mallei*. Lead pairs of host-pathogen biomarkers of infection that were identified in tissues by the combined MS methods were also readily observed in biological fluids from infected subjects. Our results demonstrate that there can be a strong correlation between proteins associated with infections of solid tissues and circulating serum. Our work further demonstrates the utility of combining a multidimensional mass spectrometric analysis of traditional histology specimens with high content protein microarrays for discovery of biomarkers that are relevant to infectious diseases.

## Materials and methods

### Ethics statement

All animal research was conducted under a protocol (AP-12-022) approved by the Institutional Animal Care and Use Committee (IACUC) at USAMRIID, in compliance with the Animal Welfare Act, PHS Policy, and other Federal statutes and regulations relating to animals and experiments involving animals. The facility where this research was conducted is accredited by the Association for Assessment and Accreditation of Laboratory Animal Care, International and adheres to principles stated in the Guide for the Care and Use of Laboratory Animals, National Research Council, 2011. All animal housing areas were continuously monitored for temperature and humidity using a state-of-the-art monitoring system; results were displayed and assessed regularly to ensure animal health and welfare. The USAMRIID Veterinary Medicine Division provided quality animal care, including housing, feeding, and environmental enrichment, for all animals 24 hours a day, 7 days a week for the duration of the study, in accordance with the recommendations of the Weatherall report, “The use of non-human primates in research”. The staff included American College of Laboratory Animal Medicine boarded veterinarians who are experts in the field of laboratory animal care and use. All animals were examined and evaluated twice per day by trained study personnel. Early endpoint criteria, as specified by the score parameters within the “Post-exposure observations” section of the approved protocol, were used to determine when animals should be humanely euthanized to ameliorate any suffering.

### Tissue preparation

All animal research was conducted under an IACUC approved protocol in compliance with the Animal Welfare Act, PHS Policy, and other Federal statutes and regulations relating to animals and experiments involving animals. The facility where this research was conducted is accredited by the Association for Assessment and Accreditation of Laboratory Animal Care, International and adheres to principles stated in the Guide for the Care and Use of Laboratory Animals, National Research Council, 2011. All non-human primate studies were performed in BSL3 containment suites at the United States Army Medical Research Institute of Infectious Diseases. Animals were exposed to a targeted inhalation dose of 3 × 10^7^ colony forming units of *B. mallei* (strain FMH23344). Lung and skin tissue samples from *Chlorocebus aethiops* (African green monkeys) were soaked in a 10% neutral buffered formalin solution for 21 days and sera were γ-irradiated prior to removal from the BSL3 suite. FFPE tissue was partitioned in 5 μm-thick sections using a Leica RM2255 microtome (Leica Microsystems, Buffalo Grove, IL) and deposited onto an indium tin oxide-coated conductive glass slide (Delta Technologies Ltm, Loveland, CO). Samples for IMS were prepared from a modified protocol as previously described [16]. Briefly, paraffin wax was removed with two 100% xylene washes (5 minutes) and the tissue was gradually hydrated in successive graded ethanol washes (100% twice, 95%, and 70% for 3 minutes each). Finally the tissue was washed twice in HPLC grade water (3 minutes each). Antigen retrieval was performed in a decloaking chamber (BioCare Medical LLC, Concord, CA) by heating the slides to 105°C for 20 minutes in 10mM Tris HCl pH 7.5, under 5 psi pressure. The slides were allowed to cool to <55°C then removed from the decloaking chamber, gently rinsed with HPLC grade water, and allowed to dry (20°C).

### Histology and immunohistochemistry

Adjacent tissue sections for each tissue were stained with hematoxylin and eosin for general histology. For immunohistochemistry, polyclonal antibodies for *B. mallei* were generated in-house. Briefly, rabbits were immunized with 400 μg of formalin-killed whole cell *B. mallei*. Two booster immunizations of 400 μg were given 1 month and 4 months after the primary immunization. Rabbits were bled 2 years after the initial immunization and the antibody containing serum was isolated. FFPE tissue sections were placed on superfrost plus slides (Thermo), deparaffinized, rehydrated, blocked with methanol hydrogen peroxide for 30 minutes, and rinsed in phosphate-buffered saline (PBS), pH 7.4, for immunohistochemistry. Serum-free CAS-Block (Life Technologies, Grand Island, NY) containing 5% goat serum (Vector Labs, Burlingame, CA) was applied to the slide for 30 minutes. The polyclonal *B. mallei* antibody was diluted 1:500 in PBS and incubated with the tissue (60 min, 20°C). After washing, polymer labeled horseradish peroxidase anti-rabbit secondary antibody (Dako, Carpinteria, CA) was applied for 30 minutes, rinsed, counter stained with hematoxylin, dehydrated, and cover-slipped with Permount (Thermo, Hampton, NH). Whole slide images were scanned for analysis and manipulation using an Aperio Scanscope CS (Leica Biosystems) to assess lesions.

### On-tissue digestion and matrix application

Trypsin (40 μg) was dissolved in 500 μL of (5% acetonitrile, 10mM ammonium bicarbonate pH 8.0) to achieve a final concentration of 80 ng/μL. The diluted trypsin solution was automatically sprayed onto processed tissue sections with an ImagePrep sprayer (Bruker, Billerica, MA) using the default trypsin application method until all 500 μL was deposited onto the slide (approximately 45 minutes). The slide was allowed to dry completely between trypsin deposition iterations. The slide was placed into a petri dish with moistened blotting paper to maintain humidity, and incubated for 12 hrs (37°C). After digestion, a 1:10 dilution of calibration solution #1 (Applied Biosystems, Grand Island, NY) was mixed 1:1 with α-cyano-4-hydroxycinnamic acid [CHCA] (7 mg/mL in 50% acetonitrile/0.5% TFA) and 0.4 μL was spotted around the four corners of the tissue and allowed to dry. Finally, CHCA was sprayed over the slide using the automatic TM-Sprayer (HTX Technologies, Carrboro, NC) as described previously [30].

### Imaging mass spectrometry

IMS experiments were performed using an AB Sciex 5800 MALDI TOF-TOF (Applied Biosystems), with an Nd:YAG laser operating at 349 nm and a fixed laser beam diameter of 75 μM. Processed slides coated with CHCA were placed into a microscope slide adaptor to be used with the stainless steel carriage (LaserBio Labs, Sophia-Antipolis Cedex, France). To bridge conductivity between the slide and the carriage, copper tape (Electron Microscope Sciences, Hatfield, PA) was used to fix the slides to the MALDI plate. Prior to imaging, a plate map and internal calibration were performed using Calmix1 (Applied Biosystems). For data acquisition, 4800 imaging software (Applied Biosystems) was used to automatically raster the laser in 75 μm in both the X and Y directions. MS1 analysis was performed in reflector positive mode, scanning 500–2500 m/z with a focus mass of 1250 m/z. For each ‘pixel’, 50 laser shots were fired at 1000Hz.

### Imaging data analysis

Disk image files (.img) recorded by the 4800 Imaging Software were opened for analysis in TissueView V1.1 (Applied Biosystems). Mass spectrometry images were extracted and normalized to total ion current (TIC). The IMS spectra were digitally superimposed on IHC imaged slides by using Adobe Photoshop CS3 (Adobe Systems Incorporated, San Jose, CA).

### Laser capture microdissection

The FFPE sections were fixed onto 1.0 PEN membrane slides (Zeiss, Jena, Germany) and stained with Mayer’s hematoxylin to aid in visualization, as described previously [31]. Regions of interest containing bacteria or regions free from infection were identified and targeted for dissections by the Arcturus laser capture microdissection system (Life Technologies). Dissected tissue was captured on Adhesive Caps (Zeiss) for further processing. One spot from the region of interest was cut from five adjacent tissue sections and pooled for analysis. The micro-dissected tissue was fragmented with trypsin, using a LiquidTissue digestion kit (Expression Pathology, Rockville, MD) according to the manufacturer’s directions.

### Protein digestion in solution

A lysate from purified and irradiated *B. mallei* was prepared as described previously [32]. Peptides from recombinant GroEL from *B. mallei* (Uniprot ID#: Q9F712) were produced as previously described [31]. Both recombinant GroEL and *B. mallei* whole cell lysate were subjected to tryptic digestion in-solution according to the manufacturer’s instructions (Thermo). Briefly, 10 μL of a 1 μg/μL protein solution was mixed with 15 μL of 50mM ammonium bicarbonate pH 8.0 containing 50 mM dithiothreitol, and heated to 95°C for 5 minutes. The samples were cooled (20°C), 3 μL of a 100mM iodoacetamide solution was added and incubated 20 minutes (20°C) in the dark. Finally, 1 μL of a 100ng/μL trypsin solution (50 mM ammonium bicarbonate, pH 8.0) was added and incubated for 12 h (30°C). The tryptic peptides were desalted by passing over C18 spin columns, according to the manufacturer’s protocol (Thermo).

### LC MS/MS mass spectrometry analysis

Unless otherwise noted all peptides were analyzed by LC-MS/MS on a Thermo Fisher Orbitrap ELITE (Thermo). On line liquid chromatography was performed on a Dionex Ultimate 3000 RSLnano pump (Thermo). Peptides were separated (35°C) on a 15 cm × 75 μm ID HPLC column packed with 3 μm C18 particles 100Å (Thermo), using a 135 minute gradient of 5-90% acetonitrile with 0.1% formic acid. Orbitrap MS1 scans were performed at a resolution of 120,000, with a scan range of 350–2000 m/z, and 20 MS2 data-dependent spectra were simultaneously acquired in the low resolution linear ion trap (top 20 method). The minimum signal required to trigger a data dependent scan was 5000. Collision induced dissociation (CID) was used to generate MS2 spectra with the following settings: normalize collision energy 35, activation Q 0.25, and activation time 20ms. A lock mass of 445.120030 was used to improve mass accuracy.

### In-solution digestion mass spectrometry: *B. mallei* whole cell digest/peptide library

Peptides generated from *B. mallei* whole cell lysate were analyzed using the Orbitrap and were searched against a FASTA file specific for *Burkholderia* (Tax ID: 32008) using SEQUEST HT in Proteome Discoverer 1.4. Variable modifications were set for carbamidomethylation, +57.02 Da; N-terminal acetylation, +42.01 Da; oxidation of methionine, +15.99 Da; and phosphorylation of STY, +79.97 Da. Minimum peptide length was specified to 6 amino acids and the maximum number of missed cleavages set to two. The minimal MS1 mass tolerance was set to 10 ppm and the fragment mass tolerance was set to 0.6 Da. A false discovery rate was calculated using PERCOLATOR and was set at <1% to score high-confidence peptide identifications, and significant protein identification was based on inclusion of two or more high-confidence peptide identifications.

### Laser capture microdissection mass spectrometry: global host protein identification

Peptides from generated from LCM were analyzed by LC-MS/MS on a Thermo Fisher Orbitrap ELITE (Thermo). Data generated from the orbitrap was searched against a FASTA file specific for the Old World Monkey proteome (Cercopithecidae: Tax ID 9567) using SEQUEST HT in Proteome Discoverer 1.4. Variable modifications were set for carbamidomethylation, +57.02 Da; N-terminal acetylation, +42.01 Da; oxidation of methionine, +15.99 Da; and phosphorylation of STY, +79.97 Da. Minimum peptide length was specified to 6 amino acids and the maximum number of missed cleavages set to two. The minimal MS1 mass tolerance was set to 10 ppm and the fragment mass tolerance was set to 0.6 Da. A false discovery rate was calculated using PERCOLATOR and was set at <1% to score high-confidence peptide identifications, and significant protein identification was based on inclusion of two or more high-confidence peptide identifications.

### *B. mallei* identification using PEAKS

Peptides from generated from LCM were analyzed by LC-MS/MS on a Thermo Fisher Orbitrap ELITE (Thermo). To maximize potential identifications generated from the orbitrap (.raw files), data was processed and analyzed by using PEAKS 7.0 (Bioinformatics Solutions Inc., Waterloo, Canada) due to its proprietary method that involves both de novo sequencing and database searching against the swissprot/reviewed FASTA file for *Burkholderia* [tax ID: 32008]. Variable modifications were set for carbamidomethylation, +57.02 Da; N-terminal acetylation, +42.01 Da; oxidation of methionine, +15.99 Da; and phosphorylation of STY, +79.97 Da. Minimum peptide length was specified to 6 amino acids and the maximum number of missed cleavages set to two. The minimal MS1 mass tolerance was set to 10 ppm and the fragment mass tolerance was set to 0.6 Da. A false discovery rate of <1% was used to score high-confidence peptide identifications from a database search and a *de novo* identification must have an ALC% >80 to be considered for analysis.

### Recombinant GroEL analyzed by MALDI TOF TOF

Peptides from recombinant GroEL from *B. mallei* (Uniprot ID#: Q9F712) were analyzed on an AB Sciex 5800 MALDI TOF-TOF in MSMS reflector positive mode. Prior to MSMS analysis a survey MS1 scan was first taken. Peaks from the survey scan were interpreted so that MSMS data was acquired on the strongest precursors first (up to 20). This methodology was used for three different interpretation ranges: 900–1500, 1500–2000, and 2000–3500 m/z. A survey peak with a signal to noise less than 35 was ignored. The MSMS spectra were collected by firing 1000 laser shots at 1000Hz. MS2 collision energy was set to 1kV and collision induced dissociation was used with atmospheric air as the collision gas. The MSMS data files were exported from Data Explorer (Applied Biosystems) as .t2d files and processed using MASCOT distiller (Matrix Science, Boston, MA) into MGF files, then searched against a FASTA file specific to the *Burkholderia mallei* proteome [tax ID: 320389], using SEQUEST HT in Proteome Discoverer 1.4 (Thermo Fisher). Variable modifications were set for carbamidomethylation, +57.021 Da; N-terminal acetylation, +42.011 Da; oxidation of methionine, +15.995 Da; and phosphorylation of Ser, Thr or Tyr, +79.966 Da. Minimum peptide length was specified to 6 amino acids and the maximum number of missed cleavages set to two. The minimal MS1 mass tolerance was set to 50 ppm and the fragment mass tolerance was set to 0.6 Da. Only peptides corresponding to GroEL/Q9F712 with Xcorr values >2.0 for +1 charge state were considered for analysis.

### Label free quantitation of host proteins

A region that stained negative for the presence of *B. mallei* and the diseased abscess were isolated and pooled using LCM. Peptides generated from these regions were analyzed by LC-MS/MS on a Thermo Fisher Orbitrap ELITE (Thermo) in triplicate. To quantitatively compare host protein changes in the two regions, the triplicate runs were grouped and the data were processed and analyzed by using PEAKS 7.0 (Bioinformatics Solutions Inc., Waterloo, Canada) against the swissprot/reviewed FASTA file for the Old World Monkey proteome (Cercopithecidae: Tax ID 9567). Variable modifications were set for carbamidomethylation, +57.02 Da; N-terminal acetylation, +42.01 Da; oxidation of methionine, +15.99 Da; and phosphorylation of STY, +79.97 Da. Minimum peptide length was specified to 6 amino acids and the maximum number of missed cleavages set to two. The minimal MS1 mass tolerance was set to 10 ppm and the fragment mass tolerance was set to 0.6 Da. A false discovery rate of <1% was used to score high-confidence peptide identifications from a database search and a de novo identification must have an ALC% >80 to be considered for analysis. For quantitation all runs were first normalized to total ion current. A protein was considered quantitatively different if it met the following conditions: 1) more than one peptide was identified from it, 2) the protein must be detectable in all three technical replicates (triplicate runs), and 3) the fold change must be greater +/− 2.0. Label-free quantitation was performed by peak area integration normalized to total ion current for each peptide identified. Proteins were considered to be significantly different between samples based on a −10 log P score of 20 (equivalent to a *p* value of 0.01).

### Recombinant protein microarray

Host antibody responses to GroEL were examined in a microarray format (manuscript in preparation). The microarray consisted of >300 recombinant *Burkholderia* proteins and controls. The protein targets selected for this microarray were essential enzymes, virulence factors, and vaccine candidates. The proteins were produced in an *E. coli* expression system and affinity purified using tags present in the recombinant proteins. Briefly, genes encoding the *Burkholderi*a proteins, including GroEL, and control proteins from *Yersinia pestis* (hupB, y1030; bolA, y1025) were cloned into pEXP7-DEST, a glutathione-S-transferase, T7-based *E. coli* expression vector (Life Technologies). Soluble proteins were affinity purified using GSTrap HP columns on an FPLC chromatography system (GE Healthcare Life Sciences, Piscataway, NJ). The purified proteins were characterized for correct molecular weight and purity using an Agilent Bioanalyzer 2100 (Agilent Technologies, Santa Clara, CA). The protein microarray was produced by spotting purified recombinant proteins (0.1 mg/ml) in 4 replicates on nitrocellulose-coated slides (Maine Manufacturing, Sanford, ME), using an inkjet printer (Arrayjet North America, Cambridge, MA). The printed spots were approximately 120 μm in diameter. The microarray was probed with sera from the *B. mallei* infected animals. The slides were first blocked with a blocking buffer (PBS, 0.1% Tween-20, and 3% BSA). Sera were diluted 1:150 (PBS containing 3% BSA and 0.2% Tween 20) and incubated on the microarray surface. Antibody binding was detected by adding anti-human IgG-Alexafluor 647 (1:1000) conjugate (Life Technologies). The slides were dried and scanned by GenePix 4000B laser scanner (Molecular Devices, Sunnyvale, CA), and image analysis was performed using GenePix Pro 6.0 software.

### Calprotectin ELISA

The abundance of calprotectin in the serum was examined using a commercially available Calprotectin (Serum) ELISA kit from DRG International, Inc. (Springfield, NJ, USA). The assay was performed according to the manufacturer’s protocol. Serum samples were diluted 1:50 (*Chlorocebus aethiops* only) or 1:100 (all others) with wash buffer prior to analysis. Quantitation of calprotectin within the serum samples was calculated by generating a standard curve with recombinant human calprotectin that was provided in the kit. A 4-parameter-algorithm was used to fit the data generated from the standard curve [Concentration (semi-log) vs. Absorbance at 450nm] and calculate unknown concentrations from raw absorbance values. A two-tailed Student’s *T* test was used to determine statistical significance, using *p* ≤ 0.05 as the acceptance criterion.

## Additional file

Additional file 1: Table S1. Detailed list of proteins identified by MS.

## Abbreviations

IMS: Imaging mass spectrometry
MALDI: Matrix assisted laser desorption ionization
FFPE: Formalin-fixed paraffin embedded
TOF: Time of flight
IHC: Immunohistochemistry
